# Stratification for RRMM and Risk-Adapted Therapy: Sequencing of Therapies in RRMM

**DOI:** 10.3390/cancers13235886

**Published:** 2021-11-23

**Authors:** Georg Jeryczynski, Arnold Bolomsky, Hermine Agis, Maria-Theresa Krauth

**Affiliations:** 1 Division of Oncology, Department of Medicine I, Medical University of Vienna, 1090 Vienna, Austria; georg.jeryczynski@meduniwien.ac.at; 2 Lymphoid Malignancies Branch, Center for Cancer Research, National Cancer Institute, National Institutes of Health, Bethesda, MD 20892, USA; arnold.bolomsky@nih.gov; 3 Division of Hematology and Hemostaseology, Department of Medicine I, Medical University of Vienna, 1080 Vienna, Austria; hermine.agis@meduniwien.ac.at

**Keywords:** multiple myeloma, relapsed and refractory, multi-refractory, extramedullary disease, high-risk cytogenetics

## Abstract

**Simple Summary:**

In recent years, many new therapeutic options for newly diagnosed, as well as relapsed and refractory multiple myeloma (MM) have been approved. They have dramatically improved progression-free and overall survival. Despite these advances, MM still remains incurable and eventually all cases will become refractory to standard treatments. Many new substances and drug classes have become available, which has greatly increased treatment options. However, the right sequencing of novel treatments has become the main challenge in MM therapy. Guidelines can provide helpful recommendations, but diversifying treatment patterns make a one-fits-all approach impossible. This review aims to summarize existing evidence regarding the sequencing of novel agents and will also focus on groups of special interest such as elderly and frail patients.

**Abstract:**

The treatment landscape for relapsed multiple myeloma (RRMM) has experienced an unprecedented wave of innovation. Implementation of numerous new substances and drug classes with different modes of action is made possible in routine clinical practice. Next generation proteasome inhibitors, monoclonal antibodies, as well as first in class agents such as selinexor and venetoclax have widened the therapeutic spectrum. This has led to an increase in progression-free and overall survival. Consequently, new challenges for treating physicians in choosing the right treatment at the right stage of the disease have been generated. Several trials support the use of novel agents in the frontline treatment of newly diagnosed multiple myeloma. The use of lenalidomide or bortezomib as a backbone in the first-line setting, requires strategies for treatment once these patients relapse and are refractory to these drugs. Despite the variety of options, selecting the optimal treatment strategy is difficult, since multiple factors have to be considered: patient-specific factors such as age and co-morbidities, as well as myeloma/tumor specific factors such as cytogenetics and relapse kinetics. This review intends to summarize the existing data and guidelines regarding the optimal sequencing of treatments of RRMM using already approved agents as well as agents under investigation.

## 1. Introduction

**Figure 1:**
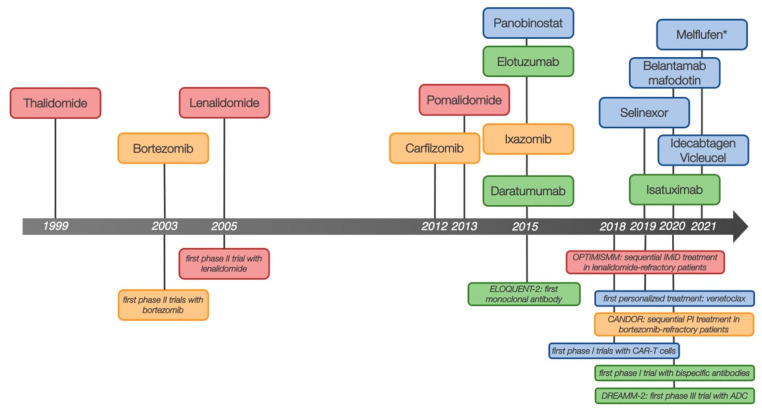
Overview of milestone trials and new agents in RRMM with year of FDA approval. Red: immunomodulators, yellow: proteasome inhibitors, green: monoclonal antibodies, blue: others substance classes; ADC—antibody drug conjugate). * As of October 2021, melflufen has been withdrawn from the US market.

After the introduction of the first-generation immunomodulatory drugs (IMiDs) and proteasome inhibitors (PIs) in the early 2000s, the treatment landscape in multiple myeloma (MM) has seen an unprecedented wave of innovation since 2015 (Figure 1). Apart from the implementation of next generation PIs (carfilzomib, ixazomib) and IMiDs (lenalidomide, pomalidomide), highly active monoclonal antibodies (mabs: daratumumab, isatuximab, elotuzumab, belantamab mafodotin) as well as novel drugs targeting different structures (e.g., selinexor) have been approved. A variety of complex immunotherapeutic strategies and concepts have been established. More recently, bispecific antibodies and chimeric antigen receptor t-cells (CAR-T) were made available and continue to improve outcome in relapsed and refractory MM (RRMM). To date, the overall survival (OS) in RRMM now can reach up to or even exceed 4 years depending on age, cytogenetic characteristics, comorbidities, previous treatment regimen and whether autologous stem cell transplant (ASCT) was performed [1,2,3,4]. Today’s challenge is to define the most appropriate treatment algorithm for each patient with respect to predefined common risk factors as well as highly individual disease characteristics. Drug sequencing and risk-adapted decision making are the most important issues now and in the near future. In this review, we attempt to address these issues.

## 2. RRMM in a Changing Treatment Landscape

Intensive first-line regimens with triplet PIs and IMiDs combinations, followed by maintenance strategies, provide the standard of care in newly diagnosed MM. Recent phase III trials in newly diagnosed transplant-eligible (NDMM-TE) and transplant-ineligible MM (NDMM-TI) introduced the anti-CD38 antibody daratumumab as a highly effective combination partner in triplet and quadruplet regimens in both NDMM-TE and NDMM-TI with only minimal additive side effects. Especially quadruplet combinations impressively increase progression-free survival (PFS) and minimal residual disease (MRD) rates which in turn may possibly result in an improved survival [5,6,7,8]. Data from the FORTE-trial also point towards a possible role of carfilzomib in NDMM-TE [9].

The incorporation of lenalidomide, bortezomib, daratumumab and, eventually, carfilzomib in the frontline treatment creates unique challenges for subsequent therapy decisions in the RRMM setting as these agents currently form the basis of many RRMM treatments.

Current guidelines and recommendations consequently recommend various triplet-therapies in early relapses [10,11,12], but offer limited evidence or guidance regarding the optimal sequencing. Head-to-head comparisons of triplet therapies involving multiple new drugs are currently not available and will remain the exception in the foreseeable future. Table 1 provides an overview of recent landmark trials in RRMM. All trials showed significantly improved outcomes for the investigational treatments. When available, MRD rates were also significantly higher. However, even as more and more data from large trials in NDMM become available, results from trials in RRMM (with only small numbers or no subjects with refractory disease included) must be carefully interpreted with caution due to limited representativeness of these patient-collectives for contemporary MM patients.

Real-world analyses can provide an important insight into this issue. An analysis of 788 patients treated with lenalidomide/dexamethasone (Rd) and 447 patients treated with bortezomib/dexamethasone (Vd) as the second-to-fourth line of therapy was published in 2019. The eligibility criteria of six published randomized controlled trials (RCTs) that used one of the two regimens as the control arm were applied to this cohort. The resulting exclusion rate in this study ranged substantially from 47.9–72.3%, highlighting the highly preselected nature of trial populations. In summary, real-world patients tended to be older, with more prior lines of therapy, more comorbidities and the disease more often refractory to the previous line of treatment. Common exclusion criteria included concomitant cancer, cardiovascular, and renal disease. Accordingly, RCT-ineligible patients had significantly worse outcome than RCT-eligible patients receiving the same treatment (3-year survival rate 63.5% vs. 74.4%, *p* = 0.02, HR 1.46 95% CI 1.03–2.07) [13].

This underlines the daily challenge in clinical decision making in a non-trial setting. Apart from a “perfect world” with clear-cut definitions of ASCT eligibility and ASCT ineligibility and clearly assigned treatment combinations, the choice of subsequent therapies depends on multiple factors, including prior treatments and patient fitness status. With an increasing number of therapeutic options, interpatient comparability decreases, while the number of cases unresponsive to various agents increases with every treatment line. Other factors such as age, renal impairment, kinetics of the relapse, and the cytogenetic risk profile further complicate treatment decisions. In the following, we aim to address these points in more detail. 

### 2.1. Lenalidomide-Refractory Disease

Thalidomide and lenalidomide have become an important back bone in many recommended induction therapies. Lenalidomide is also the preferred agent for maintenance following induction therapy and ASCT [10,11,12], as supported by large meta-analyses [41,42]. This raises the problem of IMID-refractoriness at the time of relapse. In particular, lenalidomide refractoriness is a rapidly increasing issue since most patients will have been exposed to lenalidomide as part of induction therapy or within the course of maintenance. To gain insight into the performance of lenalidomide-refractory cases, recent trials were analyzed regarding this specific patient-subgroup. Older studies with lenalidomide-comparator arms usually include only a small number of lenalidomide-exposed patients ranging from about 5% in the ELOQUENT-2 and TOURMALINE-MM1 trials to 20% in the ASPIRE trial [16,17,18]. The small numbers of subjects in these subgroups make it difficult to draw definitive conclusions. Lenalidomide-refractory subjects have thus become a focus of clinical trials in RRMM in recent years. An overview is provided in Table 2.

Sequential treatment with pomalidomide after lenalidomide was the focus of several trials. The OPTIMISMM trial compared pomalidomide/bortezomib/dexamethasone (PVd) with Vd alone. All subjects were lenalidomide-exposed and 70% lenalidomide-refractory. Lenalidomide-refractory cases treated with pomalidomide had an improved PFS of 9.53 vs. 5.59 months, with a HR of 0.65 (95%CI 0.050–0.84, *p* = 0.008). This risk reduction for progression and/or death was comparable to the one in the overall cohort (HR 0.61, 95% CI 0.49–0.77, *p* < 0.0001) as was the improvment in PFS of about 4 months (11.20 vs. 7.10 months). This effect was even more pronounced in patients with only one prior line of therapy [24,43].

Other trials included pomalidomide in the control group and the investigational arm. The antibody based ELOQUENT-3 trial added elotuzumab to a pomalidomide/dexamethasone (Pd) backbone. Elotuzumab is the first-in-class SLAMF-7 antibody and the first monoclonal antibody in MM with FDA and EMA approval [31]. Isatuximab, a second generation CD38-antibody was investigated in the ICARIA-trial, that included 93% lenalidomide-refractory cases who had at least 2 prior lines of therapy. In this subgroup, the addition of isatuximab to Pd provided the same risk reduction as in the overall cohort (HR 0.59 95% CI 0.43–0.82) [32]. Recently, data from the phase III APOLLO trial, which combined Pd with daratumumab, were published. Again, the benefit of the whole cohort was retained in the lenalidomide-refractory subgroup (HR 0.66, 95% CI 0.49–0.90) [30].

The CANDOR trial evaluated the combination of carfilzomib and dexamethasone with or without daratumumab in subjects with a median of 2 prior lines of therapy. About a third of the cases were refractory to lenalidomide. The PFS improvement in this subgroup was consistent with the overall trial population with a hazard ratio (HR) of 0.47 (95% CI 0.29–0.78). All cases were lenalidomide-exposed and 87% were considered refractory. Moreover, 70% were refractory to both lenalidomide and a PI [26].

The BELLINI trial is somewhat unique as it forms the basis for the first personalized MM treatment strategy, investigating the BCL-2 inhibitor venetoclax, that is directed against a single genetic aberration t(11;14). On the other hand, it excluded bortezomib-refractory cases. This exclusion criterion limits the applicability of the data to contemporary RRMM populations that will usually have received and frequently become unresponsive to bortezomib. Because of an excess in mortality due to infections in the intervention arm, the trial was temporarily suspended in 2019. However, in a pre-planned subgroup analysis of patients with high BCL-2 expression and positivity for the t(11;14) translocation, a clear benefit in both subgroups was seen. Subjects with high BCL-2 expression showed a 76% risk reduction for progression and/or death (HR 0.24 95% CI 0.12–0.48, *p* < 0.0001), while the t(11;14) group showed a risk reduction of 89% (HR 0.11 95% CI 0.02–0.56, *p* = 0.0040) [27]. Several trials are currently ongoing with venetoclax and other novel agents in t(11;14) positive RRMM (NCT03314181, NCT02899052, NCT03567616).

### 2.2. Bortezomib-Refractory Disease

Bortezomib has been the backbone for almost all inductions regimens for two decades. It can also be used as a maintenance therapy after ASCT. Therefore, the vast majority of patients are bortezomib-exposed upon relapse, with a substantial number of patients presenting bortezomib refractoriness. The CANDOR trial is of special interest as it evaluated the second-generation PI carfilzomib plus dexamethasone (Kd) with or without daratumumab in subjects with 1–3 prior lines of therapy (median 2) and included 29% of subjects’ refractory to bortezomib [26]. A subgroup analysis showed that the benefit in this cohort (including a small number of ixazomib-refractory cases) was less pronounced as in the total cohort (HR 0.84 95%CI 0.52–1.36 vs. 0.69 95%CI 0.57–0.83).

### 2.3. Daratumumab-Refractory Disease

Monoclonal antibodies against CD38 have become an integral part of modern myeloma treatment regimens both in the frontline as well as in the relapsed setting. Currently two anti-CD38 mabs are available: daratumumab and isatuximab. They have been evaluated in several trials in RRMM, where they have provided substantial PFS benefit for RRMM patients. Once the disease has become refractory to any anti-CD38 mab, however, new treatment options outside of clinical studies are presently limited.

This is underlined by a retrospective multicenter study from 14 centers in the US that analyzed the outcomes of anti-CD38 mab-refractory RRMM. A total of 275 subjects were included, 93.1% had received daratumumab as part of an anti-CD38 mab index regimen and 54% of subjects were considered as triple- or quad-refractory. In 249 patients with at least one line of therapy following an anti-CD38 mab (median 2, range 1–10), the ORR was 31.3%. The best outcomes were seen with PACE-like regimens (45.8%), alkylating agents (44.4%), carfilzomib (32.4%), the combination of carfilzomib with an alkylating agent (47.4%). Combination of daratumumab with an IMiD resulted in an ORR of 36.6%, while no responses were seen in combination with a PI. This result suggests a possible role for IMiDs in overcoming daratumumab refractoriness. The overall outcome in the anti-CD38 refractory cohort was poor with a median PFS of 3.4 and a median OS of 9.3 months. The best survival was observed in patients receiving daratumumab with an IMiD, or carfilzomib with an alkylating agent [44].

The activity of the daratumumab- IMiD combination is further supported by a retrospective analysis of 34 subjects treated with daratumumab, pomalidomide and dexamethasone, 22 subjects were refractory to either daratumumab, or pomalidomide or both. The rates and quality of the responses were favorable in the dara/pom-naïve cohort (ORR 92%, sCR 33%). Single refractory patients achieved an ORR of 41% and double refractory subjects 33%. However, these responses were short-lived (median PFS 5.7 months for single and 3.3 months for double refractory subjects). The median overall survival in the refractory group of patients was also substantially shorter than in the treatment-naïve cohort (treatment-naïve not reached, single refractory 15.2 months, double refractory 13.1 months) [45].

Data on retreatment with anti-CD38 mabs or treatment beyond progression are scarce. A small case series described the reintroduction of an IMiD to overcome daratumumab resistance. All six patients had received daratumumab monotherapy for at least 6 months and were refractory to at least lenalidomide or pomalidomide, four were resistant to both. Pomalidomide was added in the four subjects resistant to pomalidomide, while lenalidomide was added in the two lenalidomide-resistant patients. The time from the last use of IMiD to reintroduction ranged from 13.2 to 37.1 months. All but one patient achieved at least a minimal response (CBR 83.3%) with 50% reaching a partial response or better. The duration of response ranged from 2 to 8 months [46].

Currently, there are only limited data regarding the sequential use of daratumumab and isatuximab. In a case series of 9 subjects treated with isatuximab/pomalidomide following daratumumab treatment, all patients were IMiD-exposed, eight had received prior pomalidomide. Five patients achieved a PR and two patients had at least a minimal response [47]. Mikhael et al. reported the first results of a multicenter trial evaluating the use of isatuximab monotherapy in 32 daratumumab-refractory patients. A total of 75% were double, 28% were quad- or penta-refractory, and 60% had received daratumumab immediately prior to isatuximab. After a median exposure time of 8.3 weeks (range 1–74 weeks), only one MR was observed. However, stable disease was observed in 17 patients, 7 of which lasted for more than 6 months [48].

### 2.4. Multi-Drug Refractoriness and New Treatment Options

The current tendency to use highly intensified induction-treatment regimens with three or more drugs to achieve the best possible responses, leads to the very new problem of early multi-refractoriness. A large number of trials in RRMM provide the basis for numerous treatment options at first relapse. Subsequent relapses, however, are increasingly difficult to treat as patients become multi-exposed and multi-refractory. Consequently, double-class refractory patients, i.e., patients refractory to both IMiDs and PIs, and triple class refractory patients (refractory to IMiDs, PIs and usually an anti-CD38 antibody), are becoming increasingly common and form an important subgroup for recently published clinical trials. The SIRIUS study was an early example of a clinical trial in heavily pretreated and multi-refractory patients, with 95% of patients refractory to a PI and an IMiD, and 75% with PI, IMiD and alkylating agent refractory disease. After a median of 5 prior lines of therapy, ORRs were 29.7% and 22.8% for double and triple refractory patients, respectively [35]. Recently, a pooled analysis from the SIRIUS and the phase II GEN501 study (148 subjects) reported an ORR of 30.4% and a 3-year overall survival rate of 36.5% after a median of 5 prior lines of therapy with 87% double and 68% triple refractory cases [36].

High proportions of double refractory cases were also included in the ELOQUENT-3 (70% refractory to PI and lenalidomide), the ICARIA (71% refractory to PI and lenalidomide) and the APOLLO (42% refractory to PI and IMiD) trials, where these subgroups reached similar ORRs and HRs in comparison to the overall cohort [30,31,32].

Once the disease becomes resistant to an anti-CD38-antibody, usually daratumumab, further treatment options are limited. Currently, there are only three published phase II trials including more than 70% triple-class refractory cases: the STORM trial, the HORIZON OP-106 trial, and the DREAMM-2 trial.

The STORM trial tested 80 mg of selinexor, an oral selective inhibitor of the nuclear export (XPO1), twice weekly in combination with 20 mg dexamethasone. All patients had received at least 2 prior lines of therapy (median 5) and were exposed to bortezomib, carfilzomib, lenalidomide, pomalidomide, daratumumab and an alkylating agent, and were refractory to at least one IMiD, one PI and daratumumab. Almost all patients were refractory to carfilzomib and pomalidomide, 83% were additionally refractory to lenalidomide and 68% were penta-refractory, i.e., refractory to bortezomib, carfilzomib, lenalidomide, pomalidomide and daratumumab. The overall response rate (ORR) in this heavily pretreated cohort was 26% with a clinical benefit rate (i.e., minimal response or better) of 39%. There were no differences in the ORR between penta-refractory patients and the total cohort. The overall survival was 8.6 months [37]. Selinexor was approved by the FDA for penta-refractory MM with 4 prior lines of therapy in 2019, and by the EMA in the same setting in March 2021.

Melflufen, or melphalan flufenamide, is a first in class peptide-drug conjugate. Due to its lipophilic nature, melflufen diffuses through the cell membrane and releases melphalan as alkylating component after cleavage by intracellular aminopeptidases. Melphalan is highly hydrophilic and consequently gets trapped in the cell, where it induces irreversible DNA damage. Through this mechanism, cytotoxic melphalan levels can be achieved in the cell at much lower doses [49]. In the phase II HORIZON-OP 106 study, all subjects had to be refractory to either pomalidomide or daratumumab or both. A total of 76% were triple-class refractory cases, 87% were refractory to pomalidomide, 94% to daratumumab and 67% to bortezomib. The median number of prior lines of therapy was 5. ORR was 29% in the whole and 26% in the triple-class refractory population. The median PFS was 4.2 and 3.9 months for the whole and the triple class refractory cohort, respectively. OS was almost the same between the two groups at 11.6 and 11.2 months [50]. This has led to the approval by the FDA in patients with at least 4 prior lines of therapy and refractory disease to at least one proteasome inhibitor, one immunomodulatory agent and one anti-CD38 antibody. The ANCHOR study combines melflufen with either daratumumab or bortezomib in patients with 1–4 prior lines of therapy. First results showed promising ORRs of 70% and 60% in the daratumumab and bortezomib arm, respectively. The median PFS was 11.5 months in the daratumumab arm and not yet mature in the bortezomib arm [51].

The B-cell maturation antigen (BCMA, also known as TNFRSF17) is mainly found on mature B-cells and highly expressed on MM plasma cells, but virtually absent on other hematopoietic cells [52]. These special characteristics make BCMA an attractive target for mabs, bispecific antibodies, CAR-T cells and antibody-drug conjugates (ADC) [53]. Belantamab mafodotin or belamaf is the first humanized anti-BCMA ADC used in MM and was approved by the FDA and the EMA for patients with at least 4 prior lines of therapy and triple class refractory disease in 2020 based on the results of the DREAMM-2 trial. Belamaf was given intravenously at 2.5 and 3.4 mg/kg every three weeks in heavily pretreated MM. All patients were triple-class resistant [34]. Despite a higher response rate and a longer median PFS in the 3.4 mg/kg cohort (34.9% vs. 30.9% and 4.9 vs. 2.9 months), approval was granted for the lower dosing schedule due to increased toxicity, in particular thrombocytopenia and keratopathy.

BCMA-targeting CAR T-cells are currently being evaluated in several trials. Idecabtagene vicleucel (ide-cell, bb2121) was the first CAR-T cell product for RRMM with approval by the FDA in March 2021 and conditional market authorization by the EMA in June 2021. The approval was based on the phase II KarMMa trial, which included 128 subjects with a median of 6 prior lines of therapy. Eighty-four percent of the cohort were triple-, and 26% penta-refractory. The CAR-T cell product was evaluated at three dose levels (only four subjects were treated with lowest dose level of 150 × 10^6^ cells). ORR was high at 69% and 81% at 300 × 10^6^ and 450 × 10^6^ cells, respectively, with comparable results in all subgroups including high-risk CA, extramedullary disease (EMD) and penta-refractory disease. PFS was significantly better at the highest target dose (12.1 months at 450 × 10^6^ compared to 5.8 months at 300 × 10^6^ cells) [38].

Based on promising results from the LEGEND-2 trial [39], the CARTITUDE trial program investigates ciltacabtagene autoleucel, a dual-epitope binding CAR-T cell therapy directed against two BCMA epitopes. The Chinese multicenter LEGEND-2 study included 57 subjects with 3 prior lines of therapy and 60% double-refractory to PI and IMiDs [40], while the US-based CARTITUDE-1 trial included 97 more heavily pretreated patients (median of 6 prior lines of therapy, 41.2% penta-refractory) [39]. ORRs were high at 88% and 94.8%, respectively, with high rates of CR and MRD-negativity. In the Chinese study, the median PFS was reached at 15 months, and the median OS was not reached [40]. In the US cohort, the 12-month PFS and OS rates were 77% and 89%, respectively [39].

Despite these unprecedented responses in heavily pretreated RRMM, the future role of autologous CAR-T cell approaches remains uncertain. To date, there is no plateau in the survival curves. A meta-analysis of 15 CAR-T cell trials (14 in the relapsed setting) showed an ORR of 82% with 36% reaching CR and MRD-negativity rate of 77%. The median PFS was 10 months, indicating that even with high ORR and deep responses, relapses are frequent [54]. Further, the optimal timing for CAR T cell therapies in MM remains an open question. The phase III CARTITUDE-2 trial with a six arm-design aims to integrate ciltacabtagene autoleucel into various settings, ranging from standard risk to high risk NDMM, to patients with less than CR or early relapse after frontline therapy, to multi-drug exposed RRMM (NTC04133636).

## 3. Patient-Related Factors with Impact on Decision-Making

In addition to drug refractoriness, clinicians have to take more specific, disease and/or patient related factors into account to opt for the best treatment regimen. The most important and influencing factors are age, frailty, high risk cytogenetics, renal impairment, co-morbidities, and extramedullary disease. They should be incorporated into the decision-making-process aiming for the most effective treatment regimen which at the same time is also safe and well-tolerated.

### 3.1. High Risk Cytogenetics

Irrespective of the treatment algorithm, the genetic makeup of myeloma cells remains a major determinant of therapy responsiveness and outcome, even in the era of novel agents. There are various classifications for high-risk disease; however, they are mostly based on the cytogenetic abnormalities (CA) detected by fluorescence in situ hybridization (FISH). The international myeloma working group currently defines the presence of at least one of the following CA as high risk: del17p, t(4;14) and, t(14;16). They are present in 7%, 16% and 4% of cases, respectively, and are associated with an increased risk for disease progression or death [56]. Most trials subsequently incorporated these CA into prespecified subgroup analyses. The Mayo classification additionally recognizes t(14;20), aneuploidy, and del13 as high-risk features, which renders about 25% of cases at high risk [57]. The international staging system (ISS) that classifies NDMM into three groups according to albumin and beta-2-microglobulin levels [58] was recently revised (R-ISS) to incorporate CA and elevated LDH levels into the existing system [59]. Five-year overall survival according to the R-ISS was 82%, 62%, and 40% for R-ISS stage I, II, and III, respectively.

Although the adverse impact of CA on outcome is established, there is only limited evidence for a biomarker-guided approach. There are some data in NDMM suggesting that the negative impact of t(4;14) can be overcome by bortezomib-based induction regimens, which is of limited applicability in the setting of multi-exposed RRMM [60,61]. Most phase III trials retain the PFS benefit for the trial arm in the high-risk CA subgroups with very similar HR and ORRs. PFS remains substantially shorter in comparison to standard risk disease, sometimes even falling short of the control arm in the standard risk group [30]. This highlights the unmet need for effective treatment regimens to overcome an adverse cytogenetic profile in RRMM. A comprehensive overview of outcomes according to the cytogenetic risk profile is provided in Table 3.

### 3.2. Extramedullary Disease

Extramedullary disease (EMD) in MM can clinically be classified as either paraskeletal lesions (PS plasmocytoma), which arise directly from bone lesions with a persisting adhesion to the bone, and plasma cell masses in different soft tissue (ST-EMD) organs, seeded by hematogenous spreading. These soft tissue extramedullary lesions show an almost metastatic growth and most commonly affect lung, liver and lymph nodes [69]. PS plasmocytomas are more common at diagnosis than ST-EMD with incidence rates ranging from 7–34% and 2–4.5%, respectively. The incidence of the latter increases at relapse by up to 10% [70]. The incidence of EMD, especially at relapse, has been rising during recent years, which has led to some speculation that novel agents may trigger the formation of extramedullary masses. Two large series, however, provide no evidence that novel treatments have an impact on the incidence of EMD in RRMM [71,72]. However, increased LDH at diagnosis [71,72] as well as a higher incidence of p53 mutation [72] and EMD at baseline were identified as risk factors for the development of EMD at relapse. Prognosis in patients with EMD at relapse is dismal, in particular in patients with soft tissue EMD OS ranges from 5–12 months after occurrence of EMD [73].

Due to its rarity and heterogenous definitions, EMD is a subgroup frequently excluded, underrepresented or underreported in clinical trials. Additionally, while clinically important, the few available subgroup analyses often do not distinguish between PS plasmocytomas and ST-EMD, which makes interpreting results difficult (see Table 4). In recent phase III trials, proportions of EMD range from 8–39% (if reported at all). Subgroup analyses from the STORM [74], the ICARIA [75] and the HORIZON OP-106 [50] trial reported ORR of 18.5–50%. Unfortunately, most of these responses were short-lived with poorer mPFS in comparison to the overall cohorts. A retrospective analysis evaluated carfilzomib-based regimens in 45 subjects with EMD in the RRMM setting. Fifty-six percent had ST-EMD. General ORR was 27%, while the ORR in subjects treated with carfilzomib/lenalidomide/dexamethasone (KRd) was as high as 76% [76]. Pd showed an ORR of 30% in a phase II clinical trial that included 7.5% of patients with ST-EMD at relapse. OS was significantly shorter in comparison to the group without EMD (16 months vs. not reached, *p* = 0.002) [77].

Recent trials investigate the role of CAR-T cells in patients with EMD. The KarMMa and the LEGEND-2 trials showed remarkably high response rates of 70% and 82% in EMD RRMM, respectively [38,78]. However, the mPFS and OS of EMD in the LEGEND-2 trial was significantly shorter for EMD than for non-EMD patients demonstrating the continued poor outcome for these patients [78].

### 3.3. Renal Impairment

Renal impairment (RI) represents one of the most frequent MM-associated comorbidities, influencing therapy selection and outcome. RI is common, affecting up to 50% of MM patients at diagnosis and is associated with poorer survival. The median OS of patients with an eGFR ≥ 40 mL/min is about 51 months compared to only 17 months in patients with an eGFR < 40 mL/min. Achieving renal response is associated with improved survival [80,81]. Renal response occurs when renal impairment is reversed by treatment. For the harmonization of renal impairment and renal response categorization the IMWG has defined criteria [80]. These criteria should be used within clinical trials setting as well as in everyday clinical practice.

There are limited subgroup analyses in RRMM addressing patients with RI; however, the definition of RI differed between trials making interpretations and comparisons of the results difficult. ORRs using triplet combinations range between 56.4% (ICARIA) to up to 91.4% and 93.1% (OPTIMISMM, IKEMA) [82,83,84]. Even an improvement in OS could be demonstrated with Isa-Pd [82] (ICARIA) but also the double Kd [85]. No significant differences in adverse events were observed when compared to patients without RI.

The ICARIA trial also evaluated renal response rates. Complete renal response rate was higher with Isa–Pd (complete renal response was defined as improvement in eGFR <50 mL/min/1.73 m^2^ at baseline ≥ 60 mL (min/1.73 m^2^ in ≥1 post-baseline assessment). In patients with eGFR <50 mL/min/1.73 m^2^, the rate of achieving complete renal response was higher with Isa–Pd vs. Pd (71.9% vs. 38.1%). The median time to complete renal response was 4.1 vs. 7.3 weeks, respectively. A total of 31.3% vs. 19.0% had durable (≥60 days) complete responses (durable renal response was defined as ≥60 days). The median duration of complete renal response was 57.0 vs. 59.5 days, respectively [82].

In the IKEMA trial, the complete renal response and durable complete renal response (≥60 days) in patients with eGFR <50 mL/min/1.73 m² at baseline could be achieved. Complete renal response (CrR) occurred in 52.0% of the subjects in the Isa–Kd arm vs. 30.8% of the subjects in the Kd arm; these were durable in 32.0% vs. 7.7% patients, respectively. The median time to CrR was 4.4 (95% CI 0.4–34.0) weeks in the Isa–Kd arm and 13.9 (3.0–28.3) weeks in the Kd arm [84].

### 3.4. Elderly and Frail Patients

MM is more prevalent in the elderly: the median age at diagnosis is 69 years [86]. While survival improved in all age groups during the past decades, frail patients continue to experience poorer survival than younger patient collectives [87]. Several age-related factors impact the choice of treatment and patient outcomes: increased risk of organ dysfunction at diagnosis and during follow-up, increased number of comorbidities, increased risk of frailty, decreased resilience to treatment-related toxicity, ineligibility for some standard of care treatments, and decreased functional status [88].

Frailty is strongly associated with increased toxicity and several scoring systems are in use for better the categorization of patients frailty [89]. Despite this, this patient population remains under-represented in clinical trials [13].

Recent data from the ICARIA trial stratifies subjects into different frailty groups. Using a modified frailty score which incorporates age, modified CCI score and ECOG functional status, the overall ICARIA-MM population consisted of 28.0% frail (median age 75/76 years) and 69.4% fit/intermediate patients (median age 64/65 years). There were more frail patients in the Isa–Pd arm (31.2%) vs. Pd arm (24.8%). Although this difference is not significant (*p* = 0.2167) [90], a PFS and OS benefit in frail and fit/intermediate patients was observed: median PFS with Isa–Pd vs. Pd was 9.0 vs. 4.5 months in frail patients and 12.7 vs. 7.4 months in fit/intermediate patients. At the time of analysis, OS data were not mature but one-year OS probabilities with Isa–Pd vs. Pd were 66.9% vs. 58.8% in frail patients and 75.0% vs. 64.5% in fit/intermediate patients. Accordingly, ORR with Isa–Pd vs. Pd in frail patients was 52.1% vs. 34.2% (*p* = 0.0476), and 66.3% vs. 35.7% (*p* < 0.0001) in fit/intermediate patients. Strikingly, patients achieving a very good partial response (VGPR) or better were found at significantly higher rates with Isa–Pd vs. Pd in both the frail (29.2% vs. 2.6%, *p* = 0.0013) and the fit/intermediate group (34.7% vs. 10.7%, *p* < 0.0001). Finally, a longer time to definitive treatment discontinuation with Isa–Pd vs. Pd in frail and fit/intermediate patients was reported. Treatment discontinuation due to adverse events (AEs) in Isa–Pd vs. Pd occurred in 8.3% vs. 16.7% of frail patients and 7.0% vs. 11.7% of fit/intermediate patients. In an analysis from the IKEMA trial for the subgroup of patients ≥ 70 years, the PFS benefit and the ORR were similar to the younger patients < 70 years showing a consistent benefit in depth of response in both age groups. There was no signal for increased toxicity in elderly patients [91].

## 4. Conclusions

In a rapidly changing and increasingly diversifying treatment environment the right sequencing of therapies in RRMM is a bigger challenge than ever. While guidelines and published trial data can provide a possible treatment algorithm, the decision-making process must also rely patient centered factors such as treatment history, comorbidities, and individual disease characteristics. The treatment of RRMM remains a highly personalized process with the need for highly experienced myeloma specialists.

## Figures and Tables

**Table 1:** Landmark trials in multiple myeloma.

Trial Name	Combination	Phase	*n*	Prev. Lines of Therapy (Range)	Combination Approved by FDA/EMA	Response	Progression-Free Survival (Months)	Hazard Ratio (95% CI)	Survival Benefit	MRD Rate
PANORAMA-1 2014 [14]	PanoVd vs. Vd	3	387 vs. 381	1–3, 1: 51% vs. 52%	FDA EMA	ORR: 60.7% vs. 54.6%	11.99 vs. 8.08	0.63 (0.52–0.76), *p* < 0.0001	no [15] OS 40.3 vs. 35.8 months, HR 0.94 (0.78–1.14), *p* = 0.54	n.r.
ASPIRE 2015 [16]	KRd vs. Rd	3	396 vs. 396	1–3, median 2 (1–3)	FDA EMA	ORR 87.1% vs. 66.7% ≥VGPR 69.9% vs. 40.4%	26.3 vs. 17.6	0.69 (0.57–0.83), *p* = 0.0001	Yes [1] Median follow-up: 67.1 months OS 48.3 vs. 40.4 months, HR 0.794 (0.667–0.945), *p* = 0.0045	n.r.
ELOQUENT-2 2015 [17]	EloRd vs. Rd	3	321 vs. 325	1–3, median 2 (1–4)	FDA EMA	ORR 79% vs. 66% ≥VGPR 33% vs. 28%	19.4 vs. 14.9	0.70 (0.57–0.85), *p* < 0.001	yes [3] Minimum follow-up: 70.1 months 48.3 vs. 39.6 months, HR 0.82 (0.68–1.00), *p* = 0.0408	n.r.
TOURMALINE-MM1 2016 [18]	IxaRd vs. Rd	3	360 vs. 362	1–3, 1: 62% vs. 60%	FDA EMA	ORR 78% vs. 72% ≥VGPR 48% vs. 39%	20.6 vs. 14.7	0.74 (0.59–0.94), *p* = 0.01	no [4] Median follow-up: 85 months mOS 53.6 vs. 51.6 months, HR 0.939 (0.784 to 1.125), *p* = 0.495	n.r.
CASTOR 2016 [19]	DaraVd vs. Vd	3	251 vs. 247	≥1, median 2 (1–10)	FDA EMA	ORR: 82.9% vs. 63.2%, *p* < 0.001 ≥VGPR: 59.2% vs. 29.1%, *p* < 0.001	NR vs. 7.2	0.39 (0.28–0.53), *p* < 0.001		MRD 10-5 assessed at CR: 14% vs. 2%, *p* < 0.000001 [20]
POLLUX 2016 [21]	DaraRd vs. Rd	3	286 vs. 283	≥1, median 1 (1–11)	FDA EMA	ORR 92.9% vs. 76.4%, *p* < 0.001 ≥VGPR: 75.8% vs. 44.2%	NR vs. 18.4	0.37 (0.27–0.52), *p* < 0.001		MRD 10-5 assessed at CR: 26.2% vs. 6.4%, *p* < 0.000001 [22]
ENDEAVOR 2016 [23]	Kd vs. Vd	3	464 vs. 465	1–3, median 2 (IQR 1–2)	FDA EMA	ORR 77% vs. 63% ≥VGPR 54% vs. 29%	18.7 vs. 9.4	0.53 (0.44–0.65), *p* < 0.0001	yes [2] Median follow-up 44.3 vs. 43.7 months OS 47.8 vs. 38.8 months, HR 0.76 (0.63–0.92), *p* = 0.0017	n.r.
OPTIMISMM 2019 [24]	PVd vs. Vd	3	281 vs. 278	1–3, median 2 (IQR 1–2)	EMA	ORR 82.2% vs. 50.0% ≥VGPR 52.7% vs. 18.3%	11.2 vs. 7.1	0.61 (0.49–0.77), *p* < 0.0001		n.r.
BOSTON 2020 [25]	SVd vs. Vd	3	195 vs. 207	1–3, median 2 (IQR 1–2)	FDA	ORR 76.4% vs. 62.3% ≥VGPR 44.6% vs. 32.4%	13.93 vs. 9.46	0.70 (0.53–0.93), *p* = 0.0075		MRD 10-5 assessed at CR or better: 5% vs. 4%
CANDOR 2020 [26]	DaraKd vs. Kd	3	312 vs. 154	1–3, median 2 (IQR 1–2)	EMA FDA	ORR 84% vs. 75%, *p* = 0.008 ≥VGPR 69% vs. 49%	NR vs. 15.8	0.63 (0.46–0.85), *p* = 0.0027		MRD 10-5 at 12 months: 18% vs. 4%, *p* < 0.0001
BELLINI 2020 [27]	VenVd vs. Vd	3	194 vs. 97	1–3, 1: 47% vs. 45%		ORR t(11;14): 90% vs. 47% *p* = 0.0038 BCL2 high: 85% vs. 75%, *p* = 0.367 ≥VGPR t(11;14) 70% vs. 27%, *p* = 0.016 BCL2 high: 71% vs. 28%, *p* = 0.00013	t(11;14): NR vs. 9.5 BCL2 high: 22.4 vs. 9.9	t(11;14): 0.11 (0.02–0.56), *p* = 0.0040; BLC2 high 0.24 (0.12–0.48), *p* < 0.0001		MRD 10-5 assessed at CR or sCR: 13% vs. 1%, *p* = 0.00066 t(11;14): 25% vs. 0%, *p* = 0.056 BCL 2 high: 18% vs. 0%, *p* = 0.025
IKEMA 2021 [28]	IsaKd vs. Kd	3	179 vs. 123	1–3, median 2 (IQR 1–3)	FDA EMA	ORR 86.6% vs. 82.9% ≥VGPR 72.6% vs. 56.1%	NR vs. 19.15	0.531 (0.318–0.889), *p* = 0.0007		MRD 10-5 assessed at VGPR or better: 30% vs. 13%, *p* = 0.0004
PLEIADES 2020 [29]	RRMM Arm: s.c.D-Rd	2	65	≥1, median 1 (1–5)		ORR 93.8% ≥VGPR 78.5%				MRD 10-5: 15.4%
APOLLO 2021 [30]	DaraPd vs. Pd	3	151 vs. 153	≥1, median 2 (1–5)	FDA EMA	ORR 69% vs. 46% ≥VGPR 51% vs. 20%	12.4 vs. 6.9	0.63 (0.47–0.85), *p* = 0.00018		MRD 10-5 assessed at CR or sCR: 9% vs. 2%, *p* = 0.010
ELOQUENT-3 2018 [31]	EloPd vs. Pd	2	60 vs. 57	≥2, median 3 (2–8)	FDA EMA	ORR 53% vs. 26% ≥VGPR 33% vs. 18%	10.3 vs. 4.7	0.54 (0.34–0.86), *p* = 0.008		n.r.
ICARIA 2019 [32]	Isa-Pd vs. Pd	3	154 vs. 153	≥2, median 3 (IQR 2–4)	FDA EMA	ORR 60% vs. 35% ≥VGPR 32% vs. 9%	11.53 vs. 6.47	0.596 (0.436–0.0814), *p* = 0.001		MRD 10-5 assessed at CR or if clinically indicated: 5% vs. 0%
HORIZON OP-106 2020 [33]	Single arm melflufen	2	157	≥2, median 5 (2–12)	FDA	total cohort ORR 29%, ≥VGPR: 12% triple class refractory 26%, ≥VGPR: 11%	Total cohort: 4.22 Triple class refractory: 3.9			n.r.
DREAMM-2 2020 [34]	Belantamab-mafodotin 2.5 mg/kg vs. 3.4 mg/kg	2	97 vs. 99	≥3, median 6 vs. 7 (3–21), more than 4: 84% vs. 83%	FDA EMA	ORR 31% vs. 34% ≥VGPR 19% v.s 20%	2.9 vs. 4.9			n.r.
SIRIUS 2016 [35]	Dara-d	2	106	≥3, median 5 (2–14)	FDA EMA	ORR 29.2% ≥VGPR 12.3%	3.7			n.r.
Usmani et al., 2020 [36]	Dara-d pooled analysis from GEN501 and SIRIUS trials	2	148	median 5 (4–7)	FDA EMA	ORR: 30.4% ≥VGPR 14%	20.5			n.r.
STORM 2019 [37]	Single Arm Selinexor	2	122	median 7 (3–18)	FDA EMA	≥PR 26%, ≥MR 39% ≥VGPR 6.6%	3.7			n.r.
KarMMa 2021 [38]	Ide-cel CAR T cells	2	128	≥3, median 6 (3–16)	FDA EMA	ORR: total cohort 73%, ≥VGPR: 65% 300 × 10^6^: 69%, ≥VGPR: 42% 450 × 10^6^: 81%, ≥VGPR: 53%	Total cohort: 8.8 300 × 10^6^: 5.8 450 × 10^6^: 12.1			MRD 10-5 at CR or better: total cohort: 26% 300 × 10^6^: 24% 450 × 10^6^ 28%
Cartitude-1 2021 [39]	Cilta-cel CAR T cells	2	97	Media 6 (IQR 4–8)		ORR 97% ≥VGPR 93%	Not reached, 12 months PFS rate 77%			MRD 10-5 in 57 evaluable patients: 93%
Legend-2 [40]	Cilta-cel CAR T cells	1	57	Median 3 (1–9)		ORR 88% ≥VGPR 74%	15			MRD 10-4: 63%

Abbreviations: CAR T—chimeric antigen receptor, CI—confidence interval, Cilta-cel—ciltacabtagene autoleucel, CR—complete response, d—dexamethasone, Dara—daratumumab, Elo—elotuzumab, EMA—european medicines agency, FDA—food and drug association, HR—hazard ratio, Ide-cel—idecabtagen vicleucel, IQR—interquartile range, Isa—isatuximab, Ixa—ixazomib, K—Carfilzomib, MRD–minimal residual disease, NR—not reached, n.r.—not reported, ORR—overall response rate, OS—overall survival, P—pomalidomide, Pano—panobinostat, PFS—progression-free survival, PI—proteasome inhibitor, PR—partial response, R—lenalidomide, S—selinexor, sCR—stringend complete response, V—bortezomib, Ven—venetoclax, VGPR—very good partial response. Treatment-related factors with impact on decision making.

**Table 2:** Recent trials including patients refractory to prior therapy lines.

Clinical Trial including Bortezomib-Refractory Patients					
Trial	Combination	Phase	*n*	Bortezomib-Refractory	Response
SIRIUS 2016 [35]	Dara–d	2	106	90%	ORR 27.4%
ICARIA 2019 [32]	IsaPd vs. Pd	3	154 vs. 153	PI refractory: 76%	HR 0.58 (0.41–0.82)
CANDOR 2020 [26]	DaraKd vs. Kd	3	312 vs. 154	29% *	Refractory to bortezomib or ixazomib HR 0.84 (0.52–1.36)
HORIZON OP-106 2020 [50]	Single arm Melflufen	2	157	64%	n.r.
DREAMM-2 2020 [34]	Belantamab–mafodotin 2.5 mg/kg vs. 3–4 mg/kg	2	97 vs. 99	76%	ORR 29.7% vs. 31.1%
APOLLO 2021 [30]	Dara-Pd vs. Pd	3	151 vs. 153	PI refractory 48%	HR 0.73 (0.49–1.08)
**Clinical Trials including Lenalidomide-Refractory Patients**					
**Trial**	**Combination**	**Phase**	***n***	**Lenalidomide Refractory**	**Response**
SIRIUS 2016 [35]	Dara–d	2	106	88%	ORR 28%
ENDEAVOR 2016 [23]	Kd vs. Vd	3	464 vs. 465	25.3%	HR 0.80 (0.57–1.11)
ELOQUENT-3 2018 [31]	EloPd vs. Pd	2	60 vs. 57	87%	Double refractory: HR 0.56 (0.33–0.97)
ICARIA 2019 [32]	IsaPd vs. Pd	3	154 vs. 153	93%	HR 0.59 (95% CI 0.43–0.82)
OPTIMISMM 2019 [24]	PVd vs. Vd	3	281 vs. 278	70%	HR 0.65 (95% CI 0.50–0.84)
CANDOR 2020 [26]	DaraKd vs. Kd	3	312 vs. 154	33%	HR 0.47 (95% CI 0.29–0.78)
BELLINI 2020 [27]	VenVd vs. Vd	3	194 vs. 97	22%	n.r.
DREAMM-2 2020 [34]	Belantamab–mafodotin 2.5 mg/kg vs. 3.4 mg/kg	2	97 vs. 99	89%	ORR 29.9% vs. 35.2%
APOLLO 2021 [30]	Dara-Pd vs. Pd	3	151 vs. 153	80%	HR 0.66 (0.49–0.90)
IKEMA 2021 [28]	IsaKd vs. Kd	3	179 vs. 123	32.8%	HR 0.58 (0.35–0.96)
**Clinical Trials including Double and Triple Class Refractory Patients**					
**Trial**	**Combination**	**Phase**	***n***	**Refractoriness to Classes**	**Response**
SIRIUS 2016 [35]	Dara–d	2	106	Double (PI + IMiD): 95% Triple (PI + IMiD +alkylating agent): 75%	ORR 29.7% ORR 22.8%
EQUULEUS (MMY1001) 2017 [55]	DaraPd–arm	1b	103	Double (PI + IMiD): 71%	ORR 57.5%
ELOQUENT-3 2018 [31]	EloPd vs. Pd	2	60 vs. 57	Double (PI + Lenalidomide) 70%	HR 0.56 (0.33–0.97)
Usmani et al., 2020 [36]	Dara–d pooled analysis from GEN501 and SIRIUS trials	2	148	Double (PI + IMiD) 87% Triple: 68%	n.r.
STORM 2019 [37]	Single arm Selinexor	2	122	Triple (≥1 Imid, ≥1 PI, Daratumumab): 100%	ORR 39%, PR or better: 26%
ICARIA 2020 [32]	IsaPd vs. Pd	3	154 vs. 153	Double (PI + Lenalidomide): 71%	HR 0.58 (0.40–0.58)
HORIZON OP-106 2020 [50]	Single arm Melflufen	2	157	Triple (PI + IMiD + CD38): 76%	CBR: 39% ORR: 26% ≥VGPR: 11%
DREAMM-2 2020 [34]	Belantamab–mafodotin 2.5 mg/kg vs. 3.4 mg/kg	2	97 vs. 99	Triple (PI + IMiD + CD38 **): 100%	ORR: 30.9% vs. 34.9%
APOLLO 2021 [30]	DaraPd vs. Pd	3	151 vs. 153	Double Len + PI: 42%	HR 0.74 (0.49–1.12)
IKEMA 2021 [28]	IsaKd vs. Kd	3	179 vs. 123	Double (PI + IMiD) 21%	n.r.

* Bortezomib or ixazomib-refractory, ** anti-CD38 refractory or intolerant or both. Abbreviations: CAR T—chimeric antigen receptor, CBR—clinical benefit rate, CI—confidence interval, Cilta-cel—ciltacabtagene autoleucel, CR—complete response, d—dexamethasone, Dara—daratumumab, Elo—elotuzumab, EMA—European medicines agency, EMD—extramedullary disease, FDA—food and drug association, HR—hazard ratio, Ide-cel—idecabtagen vicleucel, IMiD—immunomodulator, IQR—interquartile range, Isa—isatuximab, Ixa—Ixazomib, K—Carfilzomib, MRD– minimal residual disease, n.r.—not reported, ORR—overall response rate, OS—overall survival, P—pomalidomide, Pano—panobinostat, PFS—progression-free survival, PI—proteasome inhibitor, PR—partial response, PS—paraskeletal, R—lenalidomide, S—selinexor, V—bortezomib, Ven—venetoclax, VGPR—very good partial response.4. Patient-related factors with impact on decision making.

**Table 3:** Response rates of recent landmark trials according to cytogenetic risk status.

Trial Name	Combination	Phase	*n*	Cytogenetics Available	Standard Risk vs. High Risk *	Response in Standard Risk Cytogenetics	Response in High Risk Cytogenetics
PANORAMA-1 2014 [14]	Pano-Vd vs. Vd	3	387 vs. 381	26%	82% vs. 18%	PFS n.r. HR: 0.88 (0.60–1.29) ORR n.r.	PFS: n.r. HR: 0.47 (0.18–1.25) ORR: n.r.
ASPIRE 2015 [16]	KRd vs. Rd	3	396 vs. 396	53%	76% vs. 24%	PFS: n.r. HR: 0.66 (0.48–0.90) ORR: n.r.	PFS: n.r. HR: 0.70 (0.43–1.16) ORR: n.r.
ELOQUENT-2 2015 [62]	EloRd vs. Rd	3	321 vs. 325	n.r.	approx. 70% vs. 20% †	PFS 19.7 vs. 16.6 months HR 0.77 (0.62–0.95), *p* = 0.0159 ORR: n.r.	PFS: 15.2 vs. 7.4 months HR 0.64 (0.43–0.98), *p* = 0.0331 ORR n.r.
Jakubowiak 2016 [63]	EloVd vs. Vd	2	77 vs. 75	45%	40% vs. 3%	HR: 0.62 (0.35–1.12)	PFS: n.r. HR: n.r. ORR: n.r.
TOURMALINE-MM1 2016 [18]	IxaRd vs. Rd	3	360 vs. 362	84%	75% vs. 25%	PFS: 20.6 vs. 15.6 months HR: 0.64 (n.r.) ORR: n.r.	PFS: 21.4 vs. 9.7 months HR: 0.54 (n.r.) ORR: n.r.
CASTOR 2016 [64]	DaraVd vs. Vd	3	251 vs. 247	71%	79% vs. 21%	PFS: 16.6 vs. 6.6 months HR: 0.26 (0.19–0.37), *p* < 0.0001 ORR: 84% vs. 62%, *p* < 0.0001, ≥VGPR: 62% vs. 28%, *p* < 0.0001 MRD 10^−5^: 11% vs. 3%, *p* = 0.0091	PFS: 12.6 vs.6.2 months HR: 0.41 (0.21–0.83), *p* = 0.0106 ORR: 85% vs. 56%, *p* = 0.0512, ≥VGPR: 59% vs. 32%, *p* = 0.1259 MRD 10^−5^: 15% vs. 0%, *p* = 0.0271
POLLUX 2016 [65]	DaraRd vs. Rd	3	286 vs. 283	77%	84% vs. 16%	PFS: NR vs. 18.6 months HR: 0.43 (0.32–0.57), *p* < 0.0001 ORR: 94 vs. 79%, *p* < 0.0001, ≥VGPR 82% Vs. 54%, *p* < 0.0001 MRD 10^−5^: 33% vs. 9%, *p* < 0.0001	PFS: 26.8 vs. 8.3 months HR: 0.34 (0.16–0.72), *p* = 0.0035 ORR: 89% vs. 68%, *p* = 0.0145, ≥VGPR 71% Vs. 29%, *p* = 0.0004 MRD 10^−5^: 26% vs. 0%, *p* = 0.0022
ENDEAVOR 2016 [66]	Kd vs. Vd	3	464 vs. 465	85%	73% vs. 27%	PFS: NR vs. 10.2 months HR 0.439 (0.333–0.578), *p* < 0.0001 ORR: 79.2% vs. 66.0%, *p* = 0.0002, ≥VGPR 58.8%% vs. 29.5%	PFS: 8.8 vs. 6.0 months HR: 0.646 (0.453–0.921), *p* = 0.0075 ORR: 72.2%% vs. 58.4%, *p* = 0.0190, ≥VGPR: 46.4% vs. 30.2%
OPTIMISMM 2019 [24]	PVd vs. Vd	3	281 vs. 278	68%	71% vs. 29%	PFS: n.r. HR: 0.67 (0.48–0.94) ORR n.r.	PFS: 8.44 vs. 5.32 months HR: 0.56 (0.35–0.90) ORR: n.r.
BOSTON 2020 [25]	SVd vs. Vd	3	195 vs. 207	90% |	47% vs. 53%	PFS: n.r. HR: n.r. ORR: n.r.	PFS: n.r. HR: 0.67 (0.45–0.98) ORR: 77.3% vs. 55.8%, *p* = 0.0008
CANDOR 2020 [26]	DaraKd vs. Kd	3	312 vs. 154	49%	68% vs. 32%	PFS: n.r. HR: 0.50 (0.28–0.90) ORR: n.r.	PFS: n.r. HR: 0.70 (0.36–1.40) ORR: n.r.
BELLINI 2020 [27]	VenVd vs. Vd	3	194 vs. 97	90%	81% vs. 19%	PFS: NR vs. 12.2 months HR: 0.54 (0.35- 0.84) ORR: n.r.	PFS: 9.0 vs. 11.4 HR: 1.21 (0.58- 2.52) ORR: n.r.
IKEMA 2021 [28]	IsaKd vs. Kd	3	179 vs. 123	88%	72% vs. 28%	PFS: NR vs. 19.45 months HR: 0·44 (0.27–0.73) ORR: n.r.	PFS: NR vs. 18.2 months HR: 0.72 (0.36–1.45) ORR: n.r.
APOLLO 2021 [30]	DaraPd vs. Pd	3	151 vs. 153	69%	65% vs. 35%	PFS: 21 vs. 7.4 months HR: 0.51 (0.32–0.81) ORR: n.r.	PFS: 5.8 vs. 4.0 months HR: 0.85 (0.49–1.44) ORR: n.r.
ELOQUENT-3 2018 [31]	EloPd vs. Pd	2	60 vs. 57	73%	68% vs. 32%	PFS: NR vs. 4.9 months HR: 0.56 (0.27–1.14) ORR: n.r.	PFS: 6.5 vs. 2.5 months HR: 0.52 (0.22–1.25) ORR: n.r.
ICARIA 2019 [67]	IsaPd vs. Pd	3	154 vs. 153	79%	75% vs. 25%	PFS: 11.6 vs. 7.4 months HR: 0.62 (0.42–0.93) ORR: 65% vs. 42.3%, *p* = 0.0012, ≥VGPR 32% vs. 9%	PFS: 7.5 vs. 3.7 months HR: 0.66 (0.33–1.28) ORR:50.0% vs. 16.7%, *p* = 0.0031, ≥VGPR 29.2% vs. 2.8%
HORIZON OP-106 2020 [33]	Single arm melflufen	2	157	80%	53% vs. 47%	PFS: 4.4 months HR: n.r. ORR: 31%	PFS: 3.1 months HR: n.r. ORR: 20%
DREAMM-2 2020 [34]	Belantamab–mafodotin 2.5 mg/kg vs. 3–4 mg/kg	2	97 vs. 99	n.r.	45%	PFS: n.r. HR: n.r. ORR: n.r.	PFS: n.r. HR: n.r. ORR: 29.3% vs. 38.3%
SIRIUS 2016 [35]	Dara–d	2	106	89%	72% vs. 21%	PFS: n.r. HR: n.r. ORR: 29.4%	PFS: n.r. HR: n.r. ORR: 20%
EQUULEUS (MMY1001) 2017 [55]	DaraPd–arm	1b	103	85%	75% vs. 25%	PFS: n.r. HR: n.r. ORR: 58.5%	PFS: n.r. HR: n.r. ORR: 59.1%
STORM 2019 ‡ [68]	Single arm Selinexor	2	200	n.r.	61% |	PFS: 4.2 months HR: n.r. ORR: 29.5%, CBR 38.5%	PFS: 3.8 months HR: n.r. ORR: 20.5%, CBR: 35.2%
KarMMa 2021 [38]	Ice-cel CAR T cells	2	128	87%	59% vs. 41%	ORR ≥ 50%	ORR ≥ 50%

* proportion of the patients with available cytogenetics. † High risk: ISS stage II or III and t(4;14) or del(17p) abnormality, standard risk: not high risk or low risk, low risk ISS stage I or II disease, an absence of t(4;14), del(17p), and 1q21 abnormalities, age < 55 years. ‡ pooled analysis from part 1 and part 2 Storm trial. | high risk defined as del(17p), t(4;16), t(4;14), 1q21 amplification. Abbreviations: CAR T—chimeric antigen receptor, CBR—clinical benefit rate, CI—confidence interval, Cilta-cel—ciltacabtagene autoleucel, CR—complete response, d—dexamethasone, Dara—daratumumab, Elo—elotuzumab, HR—hazard ratio, Ide-cel—idecabtagen vicleucel, Isa—isatuximab, Ixa—ixazomib, K—carfilzomib, MRD—minimal residual disease, NR—not reached, n.r.—not reported, ORR—overall response rate, P—pomalidomide, Pano—panobinostat, PFS—progression-free survival, PR—partial response, R—lenalidomide, S—selinexor, V—bortezomib, Ven—Venetoclax, VGPR—very good partial response.

**Table 4:** Trials reporting outcome of patients with extramedullary disease.

Trial Name	Combination	Phase	*n*	Prev. Lines of Therapy	Extramedullary Disease	PS vs. ST-EMD	Response
Short et al., 2011 [77]	Pd	2	174		7.5%	0% vs. 100% (only treatment emergent ST-EMD)	ORR 30%
Storm subgroup analysis 2019 [74]	Single arm selinexor	2	122	Median 7	22.1%	18.5 vs. 81.5%	ORR 18.5%
SIRIUS 2016 [35]	Dara–d	2	106	≥3, median 5	13%	n.r.	21.4%
Usmani 2016 [79]	Dara single agent	Joint analysis of GEN01 + Sirius	148	Median 5 (2–14)	12%	n.r.	ORR 16.7%
HORIZON OP-106 2020 [50]	Single arm melflufen	2	157	≥2, median 5	35%	n.r.	ORR 24% PFS 2.9 months (2.0–3.8)
LEGEND-2 2020 [78]	Cilta-cel CAR T cells	1	57	Median 3 (1–9)	30%	n.r.	ORR 82%
DREAMM-2 2020 [34]	Belantamab–mafodotin 2.5 mg/kg vs. 3.4 mg/kg	2	97 vs. 99	≥3, more than 4: 84% vs. 83%	20%	n.r.	ORR 9.1% vs. 5.6%
ICARIA-MM subgroup analysis 2020 [75]	IsaPd vs. Pd	3	307	≥2, 3.5 (2–13) vs. 5.5 (2–6)	8%	IsaPd: 28.6% vs. 71.4% Pd 20% vs. 80%	ORR 50% vs. 10% PFS 4.57 vs. 1.56 HR 0.219 (0.07–0.689)
Zhou et al., 2020 [76]	Carfilzomib-based regimens	Retrospective analysis	45	Median 4 (1–9)	100%	44% vs. 56%	EMD: ORR 27%, CBR 54% KRd: ORR 76% CBR 88%
KarMMa 2021 [38]	Ice-cel CAR T cells	2	128	≥3, median 6 (3–16)	39%	n.a.	ORR 70%

Abbreviations: CAR T—chimeric antigen receptor, CBR—clinical benefit rate, Cilta-cel—ciltacabtagene autoleucel, d—dexamethasone, Dara—daratumumab, HR—hazard ratio, Ide-cel—idecabtagen vicleucel, Isa—isatuximab, K—carfilzomib, n.r.—not reported, ORR—overall response rate, P—pomalidomide, Pano—panobinostat, PFS—progression-free survival, PS—paraskeletal, ST-EMD—soft tissue extramedullary disease, V—bortezomib.

## References

[1] Siegel D.S., Dimopoulos M.A., Ludwig H., Facon T., Goldschmidt H., Jakubowiak A., San-Miguel J., Obreja M., Blaedel J., Stewart A.K. (2018). Improvement in Overall Survival With Carfilzomib, Lenalidomide, and Dexamethasone in Patients With Relapsed or Refractory Multiple Myeloma. J. Clin. Oncol..

[2] Orlowski R.Z., Moreau P., Niesvizky R., Ludwig H., Oriol A., Chng W.J., Goldschmidt H., Yang Z., Kimball A.S., Dimopoulos M. (2019). Carfilzomib-Dexamethasone Versus Bortezomib-Dexamethasone in Relapsed or Refractory Multiple Myeloma: Updated Overall Survival, Safety, and Subgroups. Clin. Lymphoma Myeloma Leuk..

[3] Dimopoulos M.A., Lonial S., White D., Moreau P., Weisel K., San-Miguel J., Shpilberg O., Grosicki S., Spicka I., Walter-Croneck A. (2020). Elotuzumab, lenalidomide, and dexamethasone in RRMM: Final overall survival results from the phase 3 randomized ELOQUENT-2 study. Blood Cancer J..

[4] Richardson P.G., Kumar S.K., Masszi T., Grzasko N., Bahlis N.J., Hansson M., Pour L., Sandhu I., Ganly P., Baker B.W. (2021). Final Overall Survival Analysis of the TOURMALINE-MM1 Phase III Trial of Ixazomib, Lenalidomide, and Dexamethasone in Patients With Relapsed or Refractory Multiple Myeloma. J. Clin. Oncol..

[5] Facon T., Kumar S.K., Plesner T., Orlowski R.Z., Moreau P., Bahlis N., Basu S., Nahi H., Hulin C., Quach H. (2018). Phase 3 Randomized Study of Daratumumab Plus Lenalidomide and Dexamethasone (D-Rd) Versus Lenalidomide and Dexamethasone (Rd) in Patients with Newly Diagnosed Multiple Myeloma (NDMM) Ineligible for Transplant (MAIA). Blood.

[6] Moreau P., Attal M., Hulin C., Arnulf B., Belhadj K., Benboubker L., Bene M.C., Broijl A., Caillon H., Caillot D. (2019). Bortezomib, thalidomide, and dexamethasone with or without daratumumab before and after autologous stem-cell transplantation for newly diagnosed multiple myeloma (CASSIOPEIA): A randomised, open-label, phase 3 study. Lancet.

[7] Mateos M.V., Dimopoulos M.A., Cavo M., Suzuki K., Jakubowiak A., Knop S., Doyen C., Lucio P., Nagy Z., Kaplan P. (2018). Daratumumab plus Bortezomib, Melphalan, and Prednisone for Untreated Myeloma. N. Engl. J. Med..

[8] Voorhees P.M., Kaufman J.L., Laubach J., Sborov D.W., Reeves B., Rodriguez C., Chari A., Silbermann R., Costa L.J., Anderson L.D. (2020). Daratumumab, lenalidomide, bortezomib, and dexamethasone for transplant-eligible newly diagnosed multiple myeloma: The GRIFFIN trial. Blood.

[9] Gay F., Musto P., Rota Scalabrini D., Galli M., Belotti A., Zamagni E., Bertamini L., Zambello R., Quaresima M., De Sabbata G. (2020). Survival Analysis of Newly Diagnosed Transplant-Eligible Multiple Myeloma Patients in the Randomized Forte Trial. Blood.

[10] Dimopoulos M.A., Moreau P., Terpos E., Mateos M.V., Zweegman S., Cook G., Delforge M., Hajek R., Schjesvold F., Cavo M. (2021). Multiple myeloma: EHA-ESMO Clinical Practice Guidelines for diagnosis, treatment and follow-up(dagger). Ann. Oncol..

[11] Moreau P., Kumar S.K., San Miguel J., Davies F., Zamagni E., Bahlis N., Ludwig H., Mikhael J., Terpos E., Schjesvold F. (2021). Treatment of relapsed and refractory multiple myeloma: Recommendations from the International Myeloma Working Group. Lancet Oncol..

[12] Mikhael J., Ismaila N., Cheung M.C., Costello C., Dhodapkar M.V., Kumar S., Lacy M., Lipe B., Little R.F., Nikonova A. (2019). Treatment of Multiple Myeloma: ASCO and CCO Joint Clinical Practice Guideline. J. Clin. Oncol..

[13] Chari A., Romanus D., Palumbo A., Blazer M., Farrelly E., Raju A., Huang H., Richardson P. (2020). Randomized Clinical Trial Representativeness and Outcomes in Real-World Patients: Comparison of 6 Hallmark Randomized Clinical Trials of Relapsed/Refractory Multiple Myeloma. Clin. Lymphoma Myeloma Leuk..

[14] San-Miguel J.F., Hungria V.T., Yoon S.S., Beksac M., Dimopoulos M.A., Elghandour A., Jedrzejczak W.W., Gunther A., Nakorn T.N., Siritanaratkul N. (2014). Panobinostat plus bortezomib and dexamethasone versus placebo plus bortezomib and dexamethasone in patients with relapsed or relapsed and refractory multiple myeloma: A multicentre, randomised, double-blind phase 3 trial. Lancet Oncol..

[15] San-Miguel J.F., Hungria V.T., Yoon S.S., Beksac M., Dimopoulos M.A., Elghandour A., Jedrzejczak W.W., Gunther A., Nakorn T.N., Siritanaratkul N. (2016). Overall survival of patients with relapsed multiple myeloma treated with panobinostat or placebo plus bortezomib and dexamethasone (the PANORAMA 1 trial): A randomised, placebo-controlled, phase 3 trial. Lancet Haematol..

[16] Stewart A.K., Rajkumar S.V., Dimopoulos M.A., Masszi T., Spicka I., Oriol A., Hajek R., Rosinol L., Siegel D.S., Mihaylov G.G. (2015). Carfilzomib, lenalidomide, and dexamethasone for relapsed multiple myeloma. N. Engl. J. Med..

[17] Lonial S., Dimopoulos M., Palumbo A., White D., Grosicki S., Spicka I., Walter-Croneck A., Moreau P., Mateos M.V., Magen H. (2015). Elotuzumab Therapy for Relapsed or Refractory Multiple Myeloma. N. Engl. J. Med..

[18] Moreau P., Masszi T., Grzasko N., Bahlis N.J., Hansson M., Pour L., Sandhu I., Ganly P., Baker B.W., Jackson S.R. (2016). Oral Ixazomib, Lenalidomide, and Dexamethasone for Multiple Myeloma. N. Engl. J. Med..

[19] Palumbo A., Chanan-Khan A., Weisel K., Nooka A.K., Masszi T., Beksac M., Spicka I., Hungria V., Munder M., Mateos M.V. (2016). Daratumumab, Bortezomib, and Dexamethasone for Multiple Myeloma. N. Engl. J. Med..

[20] Mateos M.V., Sonneveld P., Hungria V., Nooka A.K., Estell J.A., Barreto W., Corradini P., Min C.K., Medvedova E., Weisel K. (2020). Daratumumab, Bortezomib, and Dexamethasone Versus Bortezomib and Dexamethasone in Patients With Previously Treated Multiple Myeloma: Three-year Follow-up of CASTOR. Clin. Lymphoma Myeloma Leuk..

[21] Dimopoulos M.A., Oriol A., Nahi H., San-Miguel J., Bahlis N.J., Usmani S.Z., Rabin N., Orlowski R.Z., Komarnicki M., Suzuki K. (2016). Daratumumab, Lenalidomide, and Dexamethasone for Multiple Myeloma. N. Engl. J. Med..

[22] Dimopoulos M.A., San-Miguel J., Belch A., White D., Benboubker L., Cook G., Leiba M., Morton J., Ho P.J., Kim K. (2018). Daratumumab plus lenalidomide and dexamethasone versus lenalidomide and dexamethasone in relapsed or refractory multiple myeloma: Updated analysis of POLLUX. Haematologica.

[23] Dimopoulos M.A., Moreau P., Palumbo A., Joshua D., Pour L., Hajek R., Facon T., Ludwig H., Oriol A., Goldschmidt H. (2016). Carfilzomib and dexamethasone versus bortezomib and dexamethasone for patients with relapsed or refractory multiple myeloma (ENDEAVOR): A randomised, phase 3, open-label, multicentre study. Lancet Oncol..

[24] Richardson P.G., Oriol A., Beksac M., Liberati A.M., Galli M., Schjesvold F., Lindsay J., Weisel K., White D., Facon T. (2019). Pomalidomide, bortezomib, and dexamethasone for patients with relapsed or refractory multiple myeloma previously treated with lenalidomide (OPTIMISMM): A randomised, open-label, phase 3 trial. Lancet Oncol..

[25] Grosicki S., Simonova M., Spicka I., Pour L., Kriachok I., Gavriatopoulou M., Pylypenko H., Auner H.W., Leleu X., Doronin V. (2020). Once-per-week selinexor, bortezomib, and dexamethasone versus twice-per-week bortezomib and dexamethasone in patients with multiple myeloma (BOSTON): A randomised, open-label, phase 3 trial. Lancet.

[26] Dimopoulos M., Quach H., Mateos M.V., Landgren O., Leleu X., Siegel D., Weisel K., Yang H., Klippel Z., Zahlten-Kumeli A. (2020). Carfilzomib, dexamethasone, and daratumumab versus carfilzomib and dexamethasone for patients with relapsed or refractory multiple myeloma (CANDOR): Results from a randomised, multicentre, open-label, phase 3 study. Lancet.

[27] Kumar S.K., Harrison S.J., Cavo M., de la Rubia J., Popat R., Gasparetto C., Hungria V., Salwender H., Suzuki K., Kim I. (2020). Venetoclax or placebo in combination with bortezomib and dexamethasone in patients with relapsed or refractory multiple myeloma (BELLINI): A randomised, double-blind, multicentre, phase 3 trial. Lancet Oncol..

[28] Moreau P., Dimopoulos M.A., Mikhael J., Yong K., Capra M., Facon T., Hajek R., Spicka I., Baker R., Kim K. (2021). Isatuximab, carfilzomib, and dexamethasone in relapsed multiple myeloma (IKEMA): A multicentre, open-label, randomised phase 3 trial. Lancet.

[29] Chari A., Rodriguez-Otero P., McCarthy H., Suzuki K., Hungria V., Sureda Balari A., Perrot A., Hulin C., Magen H., Iida S. (2021). Subcutaneous daratumumab plus standard treatment regimens in patients with multiple myeloma across lines of therapy (PLEIADES): An open-label Phase II study. Br. J. Haematol..

[30] Dimopoulos M.A., Terpos E., Boccadoro M., Delimpasi S., Beksac M., Katodritou E., Moreau P., Baldini L., Symeonidis A., Bila J. (2021). Daratumumab plus pomalidomide and dexamethasone versus pomalidomide and dexamethasone alone in previously treated multiple myeloma (APOLLO): An open-label, randomised, phase 3 trial. Lancet Oncol..

[31] Dimopoulos M.A., Dytfeld D., Grosicki S., Moreau P., Takezako N., Hori M., Leleu X., LeBlanc R., Suzuki K., Raab M.S. (2018). Elotuzumab plus Pomalidomide and Dexamethasone for Multiple Myeloma. N. Engl. J. Med..

[32] Attal M., Richardson P.G., Rajkumar S.V., San-Miguel J., Beksac M., Spicka I., Leleu X., Schjesvold F., Moreau P., Dimopoulos M.A. (2019). Isatuximab plus pomalidomide and low-dose dexamethasone versus pomalidomide and low-dose dexamethasone in patients with relapsed and refractory multiple myeloma (ICARIA-MM): A randomised, multicentre, open-label, phase 3 study. Lancet.

[33] Richardson P., Ocio E., Oriol A., Larocca A., Rodriguez Otero P., Moreb J., Bladé J., Hassoun H., Cavo M., Alegre A. (2018). OP-106 Horizon—Melflufen Therapy for RRMM Patients Refractory to Daratumumab and/or Pomalidomide; Updated Results and First Report on PFS. Blood.

[34] Lonial S., Lee H.C., Badros A., Trudel S., Nooka A.K., Chari A., Abdallah A.O., Callander N., Lendvai N., Sborov D. (2020). Belantamab mafodotin for relapsed or refractory multiple myeloma (DREAMM-2): A two-arm, randomised, open-label, phase 2 study. Lancet Oncol..

[35] Lonial S., Weiss B.M., Usmani S.Z., Singhal S., Chari A., Bahlis N.J., Belch A., Krishnan A., Vescio R.A., Mateos M.V. (2016). Daratumumab monotherapy in patients with treatment-refractory multiple myeloma (SIRIUS): An open-label, randomised, phase 2 trial. Lancet.

[36] Usmani S.Z., Nahi H., Plesner T., Weiss B.M., Bahlis N.J., Belch A., Voorhees P.M., Laubach J.P., van de Donk N., Ahmadi T. (2020). Daratumumab monotherapy in patients with heavily pretreated relapsed or refractory multiple myeloma: Final results from the phase 2 GEN501 and SIRIUS trials. Lancet Haematol..

[37] Chari A., Vogl D.T., Gavriatopoulou M., Nooka A.K., Yee A.J., Huff C.A., Moreau P., Dingli D., Cole C., Lonial S. (2019). Oral Selinexor-Dexamethasone for Triple-Class Refractory Multiple Myeloma. N. Engl. J. Med..

[38] Munshi N.C., Anderson L.D., Shah N., Madduri D., Berdeja J., Lonial S., Raje N., Lin Y., Siegel D., Oriol A. (2021). Idecabtagene Vicleucel in Relapsed and Refractory Multiple Myeloma. N. Engl. J. Med..

[39] Berdeja J.G., Madduri D., Usmani S.Z., Jakubowiak A., Agha M., Cohen A.D., Stewart A.K., Hari P., Htut M., Lesokhin A. (2021). Ciltacabtagene autoleucel, a B-cell maturation antigen-directed chimeric antigen receptor T-cell therapy in patients with relapsed or refractory multiple myeloma (CARTITUDE-1): A phase 1b/2 open-label study. Lancet.

[40] Zhao W.H., Liu J., Wang B.Y., Chen Y.X., Cao X.M., Yang Y., Zhang Y.L., Wang F.X., Zhang P.Y., Lei B. (2018). A phase 1, open-label study of LCAR-B38M, a chimeric antigen receptor T cell therapy directed against B cell maturation antigen, in patients with relapsed or refractory multiple myeloma. J. Hematol. Oncol..

[41] Gay F., Jackson G., Rosinol L., Holstein S.A., Moreau P., Spada S., Davies F., Lahuerta J.J., Leleu X., Bringhen S. (2018). Maintenance Treatment and Survival in Patients With Myeloma: A Systematic Review and Network Meta-analysis. JAMA Oncol..

[42] McCarthy P.L., Holstein S.A., Petrucci M.T., Richardson P.G., Hulin C., Tosi P., Bringhen S., Musto P., Anderson K.C., Caillot D. (2017). Lenalidomide Maintenance After Autologous Stem-Cell Transplantation in Newly Diagnosed Multiple Myeloma: A Meta-Analysis. J. Clin. Oncol..

[43] Dimopoulos M., Weisel K., Moreau P., Anderson L.D., White D., San-Miguel J., Sonneveld P., Engelhardt M., Jenner M., Corso A. (2021). Pomalidomide, bortezomib, and dexamethasone for multiple myeloma previously treated with lenalidomide (OPTIMISMM): Outcomes by prior treatment at first relapse. Leukemia.

[44] Gandhi U.H., Cornell R.F., Lakshman A., Gahvari Z.J., McGehee E., Jagosky M.H., Gupta R., Varnado W., Fiala M.A., Chhabra S. (2019). Outcomes of patients with multiple myeloma refractory to CD38-targeted monoclonal antibody therapy. Leukemia.

[45] Nooka A.K., Joseph N.S., Kaufman J.L., Heffner L.T., Gupta V.A., Gleason C., Boise L.H., Lonial S. (2019). Clinical efficacy of daratumumab, pomalidomide, and dexamethasone in patients with relapsed or refractory myeloma: Utility of re-treatment with daratumumab among refractory patients. Cancer.

[46] Gavriatopoulou M., Kastritis E., Ntanasis-Stathopoulos I., Fotiou D., Roussou M., Migkou M., Ziogas D.C., Kanellias N., Terpos E., Dimopoulos M.A. (2018). The addition of IMiDs for patients with daratumumab-refractory multiple myeloma can overcome refractoriness to both agents. Blood.

[47] Becnel M.R., Horowitz S.B., Thomas S.K., Iyer S.P., Patel K.K., Manasanch E.E., Weber D.M., Kaufman G.P., Lee H.C., Orlowski R.Z. (2020). Descriptive Analysis of Isatuximab Use Following Prior Daratumumab in Patients with Relapsed/Refractory Multiple Myeloma. Blood.

[48] Mikhael J., Belhadj-Merzoug K., Hulin C., Vincent L., Moreau P., Gasparetto C., Pour L., Spicka I., Vij R., Zonder J. (2021). A phase 2 study of isatuximab monotherapy in patients with multiple myeloma who are refractory to daratumumab. Blood Cancer J..

[49] Mateos M.V., Blade J., Bringhen S., Ocio E.M., Efebera Y., Pour L., Gay F., Sonneveld P., Gullbo J., Richardson P.G. (2020). Melflufen: A Peptide-Drug Conjugate for the Treatment of Multiple Myeloma. J. Clin. Med..

[50] Richardson P.G., Oriol A., Larocca A., Blade J., Cavo M., Rodriguez-Otero P., Leleu X., Nadeem O., Hiemenz J.W., Hassoun H. (2021). Melflufen and Dexamethasone in Heavily Pretreated Relapsed and Refractory Multiple Myeloma. J. Clin. Oncol..

[51] Ocio E.M., Efebera Y.A., Hájek R., Granell M., Maisnar V., Straub J., Eveillard J.-R., Karlin L., Ribrag V., Mateos M.-V. (2020). ANCHOR (OP-104): Melflufen Plus Dexamethasone (dex) and Daratumumab (dara) or Bortezomib (BTZ) in Relapsed/Refractory Multiple Myeloma (RRMM) Refractory to an IMiD and/or a Proteasome Inhibitor (PI)—Updated Efficacy and Safety. Blood.

[52] Carpenter R.O., Evbuomwan M.O., Pittaluga S., Rose J.J., Raffeld M., Yang S., Gress R.E., Hakim F.T., Kochenderfer J.N. (2013). B-cell maturation antigen is a promising target for adoptive T-cell therapy of multiple myeloma. Clin. Cancer Res..

[53] Shah N., Chari A., Scott E., Mezzi K., Usmani S.Z. (2020). B-cell maturation antigen (BCMA) in multiple myeloma: Rationale for targeting and current therapeutic approaches. Leukemia.

[54] Gagelmann N., Ayuk F., Atanackovic D., Kröger N. (2020). B cell maturation antigen-specific chimeric antigen receptor T cells for relapsed or refractory multiple myeloma: A meta-analysis. Eur. J. Haematol..

[55] Chari A., Suvannasankha A., Fay J.W., Arnulf B., Kaufman J.L., Ifthikharuddin J.J., Weiss B.M., Krishnan A., Lentzsch S., Comenzo R. (2017). Daratumumab plus pomalidomide and dexamethasone in relapsed and/or refractory multiple myeloma. Blood.

[56] Sonneveld P., Avet-Loiseau H., Lonial S., Usmani S., Siegel D., Anderson K.C., Chng W.J., Moreau P., Attal M., Kyle R.A. (2016). Treatment of multiple myeloma with high-risk cytogenetics: A consensus of the International Myeloma Working Group. Blood.

[57] Stewart A.K., Bergsagel P.L., Greipp P.R., Dispenzieri A., Gertz M.A., Hayman S.R., Kumar S., Lacy M.Q., Lust J.A., Russell S.J. (2007). A practical guide to defining high-risk myeloma for clinical trials, patient counseling and choice of therapy. Leukemia.

[58] Greipp P.R., San Miguel J., Durie B.G., Crowley J.J., Barlogie B., Blade J., Boccadoro M., Child J.A., Avet-Loiseau H., Kyle R.A. (2005). International staging system for multiple myeloma. J. Clin. Oncol..

[59] Palumbo A., Avet-Loiseau H., Oliva S., Lokhorst H.M., Goldschmidt H., Rosinol L., Richardson P., Caltagirone S., Lahuerta J.J., Facon T. (2015). Revised International Staging System for Multiple Myeloma: A Report From International Myeloma Working Group. J. Clin. Oncol..

[60] Avet-Loiseau H., Leleu X., Roussel M., Moreau P., Guerin-Charbonnel C., Caillot D., Marit G., Benboubker L., Voillat L., Mathiot C. (2010). Bortezomib plus dexamethasone induction improves outcome of patients with t(4;14) myeloma but not outcome of patients with del(17p). J. Clin. Oncol..

[61] Cavo M., Tacchetti P., Patriarca F., Petrucci M.T., Pantani L., Galli M., Di Raimondo F., Crippa C., Zamagni E., Palumbo A. (2010). Bortezomib with thalidomide plus dexamethasone compared with thalidomide plus dexamethasone as induction therapy before, and consolidation therapy after, double autologous stem-cell transplantation in newly diagnosed multiple myeloma: A randomised phase 3 study. Lancet.

[62] Dimopoulos M.A., Lonial S., Betts K.A., Chen C., Zichlin M.L., Brun A., Signorovitch J.E., Makenbaeva D., Mekan S., Sy O. (2018). Elotuzumab plus lenalidomide and dexamethasone in relapsed/refractory multiple myeloma: Extended 4-year follow-up and analysis of relative progression-free survival from the randomized ELOQUENT-2 trial. Cancer.

[63] Jakubowiak A., Offidani M., Pegourie B., De La Rubia J., Garderet L., Laribi K., Bosi A., Marasca R., Laubach J., Mohrbacher A. (2016). Randomized phase 2 study: Elotuzumab plus bortezomib/dexamethasone vs bortezomib/dexamethasone for relapsed/refractory MM. Blood.

[64] Weisel K., Spencer A., Lentzsch S., Avet-Loiseau H., Mark T.M., Spicka I., Masszi T., Lauri B., Levin M.D., Bosi A. (2020). Daratumumab, bortezomib, and dexamethasone in relapsed or refractory multiple myeloma: Subgroup analysis of CASTOR based on cytogenetic risk. J. Hematol. Oncol..

[65] Kaufman J.L., Dimopoulos M.A., White D., Benboubker L., Cook G., Leiba M., Morton J., Joy Ho P., Kim K., Takezako N. (2020). Daratumumab, lenalidomide, and dexamethasone in relapsed/refractory myeloma: A cytogenetic subgroup analysis of POLLUX. Blood Cancer J..

[66] Chng W.J., Goldschmidt H., Dimopoulos M.A., Moreau P., Joshua D., Palumbo A., Facon T., Ludwig H., Pour L., Niesvizky R. (2017). Carfilzomib-dexamethasone vs bortezomib-dexamethasone in relapsed or refractory multiple myeloma by cytogenetic risk in the phase 3 study ENDEAVOR. Leukemia.

[67] Harrison S.J., Perrot A., Alegre A., Simpson D., Wang M.C., Spencer A., Delimpasi S., Hulin C., Sunami K., Facon T. (2021). Subgroup analysis of ICARIA-MM study in relapsed/refractory multiple myeloma patients with high-risk cytogenetics. Br. J. Haematol..

[68] Nooka A.K., Yee A.J., Huff C.A., Vogl D.T., Gavriatopoulou M., Chari A., Moreau P., Dingli D., Cole C.E., Lonial S. (2019). Influence of Cytogenetics in Patients with Relapsed Refractory Multiple Myeloma Treated with Oral Selinexor and Dexamethasone: A Post-Hoc Analysis of the STORM Study. Blood.

[69] Bhutani M., Foureau D.M., Atrash S., Voorhees P.M., Usmani S.Z. (2020). Extramedullary multiple myeloma. Leukemia.

[70] Rosinol L., Beksac M., Zamagni E., Van de Donk N., Anderson K.C., Badros A., Caers J., Cavo M., Dimopoulos M.A., Dispenzieri A. (2021). Expert review on soft-tissue plasmacytomas in multiple myeloma: Definition, disease assessment and treatment considerations. Br. J. Haematol..

[71] Varettoni M., Corso A., Pica G., Mangiacavalli S., Pascutto C., Lazzarino M. (2010). Incidence, presenting features and outcome of extramedullary disease in multiple myeloma: A longitudinal study on 1003 consecutive patients. Ann. Oncol..

[72] Deng S., Xu Y., An G., Sui W., Zou D., Zhao Y., Qi J., Li F., Hao M., Qiu L. (2015). Features of extramedullary disease of multiple myeloma: High frequency of p53 deletion and poor survival: A retrospective single-center study of 834 cases. Clin. Lymphoma Myeloma. Leuk..

[73] Pour L., Sevcikova S., Greslikova H., Kupska R., Majkova P., Zahradova L., Sandecka V., Adam Z., Krejci M., Kuglik P. (2014). Soft-tissue extramedullary multiple myeloma prognosis is significantly worse in comparison to bone-related extramedullary relapse. Haematologica.

[74] Yee A.J., Huff C.A., Chari A., Vogl D.T., Gavriatopoulou M., Nooka A.K., Moreau P., Dingli D., Cole C.E., Lonial S. (2019). Response to Therapy and the Effectiveness of Treatment with Selinexor and Dexamethasone in Patients with Penta-Exposed Triple-Class Refractory Myeloma Who Had Plasmacytomas. Blood.

[75] Beksac M., Richardson P.G., Unal A., Corradini P., DeLimpasi S., Gulbas Z., Kerridge I., Mikala G., Neylon A., Symeonidis A. Isatuximab Plus Pomalidomide and Dexamethasone in Patients with Relapsed/Refractory Multiple Myeloma and Soft-Tissue Plasmacytomas: Icaria-Mm Subgroup Analysis. Proceedings of the European Hematology Association Congress.

[76] Zhou X., Fluchter P., Nickel K., Meckel K., Messerschmidt J., Bockle D., Knorz S., Steinhardt M.J., Krummenast F., Danhof S. (2020). Carfilzomib Based Treatment Strategies in the Management of Relapsed/Refractory Multiple Myeloma with Extramedullary Disease. Cancers.

[77] Short K.D., Rajkumar S.V., Larson D., Buadi F., Hayman S., Dispenzieri A., Gertz M., Kumar S., Mikhael J., Roy V. (2011). Incidence of extramedullary disease in patients with multiple myeloma in the era of novel therapy, and the activity of pomalidomide on extramedullary myeloma. Leukemia.

[78] Wang B., Liu J., Zhao W.-H., Chen Y.-X., Cao X.-M., Yang Y., Zhang Y.-L., Wang F.-X., Zhang P.-Y., Lei B. (2020). Chimeric Antigen Receptor T Cell Therapy in the Relapsed or Refractory Multiple Myeloma with Extramedullary Disease--a Single Institution Observation in China. Blood.

[79] Usmani S.Z., Weiss B.M., Plesner T., Bahlis N.J., Belch A., Lonial S., Lokhorst H.M., Voorhees P.M., Richardson P.G., Chari A. (2016). Clinical efficacy of daratumumab monotherapy in patients with heavily pretreated relapsed or refractory multiple myeloma. Blood.

[80] Dimopoulos M.A., Sonneveld P., Leung N., Merlini G., Ludwig H., Kastritis E., Goldschmidt H., Joshua D., Orlowski R.Z., Powles R. (2016). International Myeloma Working Group Recommendations for the Diagnosis and Management of Myeloma-Related Renal Impairment. J. Clin. Oncol..

[81] Chen X., Luo X., Zu Y., Issa H.A., Li L., Ye H., Yang T., Hu J., Wei L. (2020). Severe renal impairment as an adverse prognostic factor for survival in newly diagnosed multiple myeloma patients. J. Clin. Lab. Anal..

[82] Dimopoulos M.A., Leleu X., Moreau P., Richardson P.G., Liberati A.M., Harrison S.J., Miles Prince H., Ocio E.M., Assadourian S., Campana F. (2021). Isatuximab plus pomalidomide and dexamethasone in relapsed/refractory multiple myeloma patients with renal impairment: ICARIA-MM subgroup analysis. Leukemia.

[83] Richardson P.G., Schjesvold F., Weisel K., Moreau P., Anderson L.D., White D., Rodriguez-Otero P., Sonneveld P., Engelhardt M., Jenner M. (2021). Pomalidomide, bortezomib, and dexamethasone at first relapse in lenalidomide-pretreated myeloma: A subanalysis of OPTIMISMM by clinical characteristics. Eur. J. Haematol..

[84] Capra M., Martin T., Moreau P., Baker R., Pour L., Min C.-K., Leleu X., Mohty M., Reinoso Segura M., Turgut M. (2020). Isatuximab Plus Carfilzomib and Dexamethasone Versus Carfilzomib and Dexamethasone in Relapsed Multiple Myeloma Patients with Renal Impairment: Ikema Subgroup Analysis. Blood.

[85] Dimopoulos M., Siegel D., White D.J., Boccia R., Iskander K.S., Yang Z., Kimball A.S., Mezzi K., Ludwig H., Niesvizky R. (2019). Carfilzomib vs. bortezomib in patients with multiple myeloma and renal failure: A subgroup analysis of ENDEAVOR. Blood.

[86] SEER Cancer Stat Facts: Myeloma. https://seer.cancer.gov/statfacts/html/mulmy.html.

[87] Jones A., Bowcock S., Rachet B. (2021). Survival trends in elderly myeloma patients. Eur. J. Haematol..

[88] Cook G., Larocca A., Facon T., Zweegman S., Engelhardt M. (2020). Defining the vulnerable patient with myeloma-a frailty position paper of the European Myeloma Network. Leukemia.

[89] Engelhardt M., Domm A.S., Dold S.M., Ihorst G., Reinhardt H., Zober A., Hieke S., Baayen C., Muller S.J., Einsele H. (2017). A concise revised Myeloma Comorbidity Index as a valid prognostic instrument in a large cohort of 801 multiple myeloma patients. Haematologica.

[90] Schjesvold F.H., Richardson P.G., Facon T., Alegre A., Spencer A., Jurczyszyn A., Sunami K., Frenzel L., Min C.K., Guillonneau S. (2021). Isatuximab plus pomalidomide and dexamethasone in elderly patients with relapsed/refractory multiple myeloma: ICARIA-MM subgroup analysis. Haematologica.

[91] Facon T., Moreau P., Martin T.G., Spicka I., Oriol A., Koh Y., Lim A., Mikala G., Rosiñol L., Yağci M. (2021). MM-092: Isatuximab Plus Carfilzomib and Dexamethasone Versus Carfilzomib and Dexamethasone in Elderly Patients with Relapsed Multiple Myeloma: IKEMA Subgroup Analysis. Clin. Lymphoma Myeloma Leuk..

